# Latarjet Procedure With Distal Tibia Allograft Augmentation for Anterior Shoulder Instability in a Convulsive Patient: A Case Report

**DOI:** 10.1155/cro/4264089

**Published:** 2025-12-28

**Authors:** Ewerton Borges de Souza Lima, Arthur Cardoso Paroneto, Gabriel Ferreira Santos Vasconcelos, Leonardo Berto, Paulo Henrique Schmidt Lara, Paulo Santoro Belangero, Alberto de Castro Pochini, Benno Ejnisman, Carlos Vicente Andreoli

**Affiliations:** ^1^ Department of Orthopedics and Traumatology, Federal University of São Paulo, São Paulo, State of São Paulo, Brazil, unifesp.br

**Keywords:** bone grafting, distal tibia allograft, glenohumeral instability, Latarjet procedure, seizure disorders, shoulder dislocation

## Abstract

This case report describes the combined use of the Latarjet procedure with distal tibia allograft augmentation to treat anterior shoulder instability in a 35‐year‐old female patient with a seizure disorder. The patient had significant bipolar bone loss and coracoid fracture pseudoarthrosis. The procedure involved using both coracoid autograft and distal tibia allograft to address the glenoid bone defect. Postoperative care included a sling for 3 weeks followed by progressive physiotherapy. At 18 months postsurgery, the patient reported high satisfaction, full recovery, and no recurrence of dislocations. Radiographic evaluations confirmed proper graft positioning and bone integration. This combined technique offers a robust solution for managing complex shoulder instability cases with significant bone loss and insufficient coracoid graft.

## 1. Introduction

Anterior glenohumeral instability is a prevalent condition, frequently associated with substantial bone defects of the glenoid and humeral head, particularly in patients with seizure disorders [1]. The Latarjet procedure is widely accepted for managing anterior shoulder instability with significant glenoid bone loss; however, in selected cases, alternative approaches and graft choices may be required. When the glenoid bone defect exceeds 25% and the coracoid process is insufficient or unavailable, options such as iliac crest autograft (Eden–Hybinette procedure) or allograft may be preferred. Furthermore, in patients with a high risk of recurrence—such as those with epilepsy, high‐demand athletes, or individuals undergoing revision surgery—alternative reconstructive strategies should be considered [2].

An alternative approach using distal tibia allograft has been proposed as an alternative solution for glenoid reconstruction, offering advantages in joint reconstruction and bone integration without donor site complications seen with the iliac crest graft [2, 3]. This case report describes the combination of the Latarjet technique with distal tibia allograft augmentation for treating anterior glenohumeral instability in a patient with seizure disorder.

## 2. Case Report

**Figure 1:**
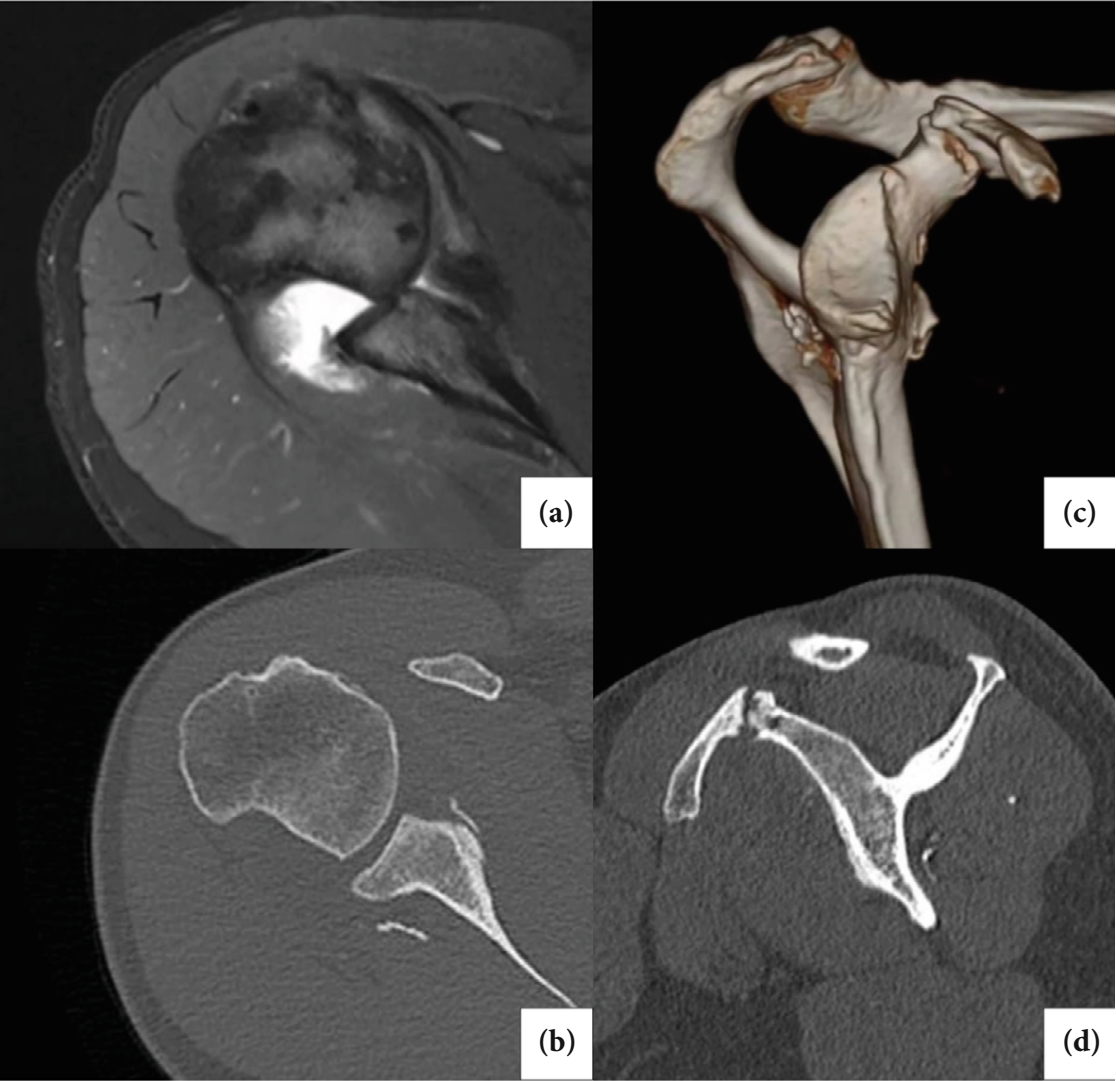
Imaging exams of the patient′s right shoulder displaying the extensive Hill–Sachs lesion, anterior glenoid bone loss, and coracoid fracture with pseudoarthrosis. (a) Axial view on magnetic resonance. (b) Axial view on computed tomography. (c) Three‐dimensional reconstruction. (d) Sagittal view on computed tomography.

A 35‐year‐old female nurse, with a history of primary dislocation of the right shoulder at age 23 during a seizure episode, reported over 100 recurrences since then. Her epilepsy was controlled with medications (lamotrigine and clobazam) and regular neurological follow‐ups. Patient presented with a positive apprehension test, limited shoulder elevation, and low shoulder function scores (as measured with the American Shoulder and Elbow Surgeons Score and the Western Ontario Shoulder Instability Index). Image exams showed extensive bipolar bone loss (Hill–Sachs lesion and bony Bankart). Furthermore, coracoid process pseudoarthrosis was identified. Key physical and imaging findings related to her glenohumeral instability are presented in Table 1 and Figure 1. Glenoid bone loss, Hill–Sachs dimensions, and glenoid track assessment were measured using three‐dimensional CT scan, following established radiological techniques [4–6].

**Table 1:** Patient′s shoulder function score, physical exam, and imaging findings before and after the surgery.

	**Pre-op**	**Post-op**
Function scores	‐ American Shoulder and Elbow Surgeons Score (ASES): 39 ‐ Western Ontario Shoulder Instability Index (WOSI): 130	‐ ASES: 83 ‐ WOSI: 48

Physical exam	‐ Positive apprehension test ‐ Positive sulcus sign ‐ Elevation: 140° (R)/170° (L) ‐ Internal rotation: T7 (bilateral) ‐ Lateral rotation: 20° (R)/70° (L) ‐ No neurovascular deficits	‐ Negative apprehension test ‐ Positive sulcus sign ‐ Elevation: 170° (bilateral) ‐ Internal rotation: T7 (bilateral) ‐ Lateral rotation: 45° (R)/70° (L) ‐ No neurovascular deficits

Imaging exam	‐ 18% glenoid bone loss ‐ Hill–Sachs lesion (2.6 × 2.2 × 0.9 cm) ‐ Off‐track lesion ‐ Coracoid process nonunion (2.1 × 0.8 cm on sagittal plane)	‐ Good positioning of the graft and synthesis material ‐ Signs of consolidation

Due to significant bipolar bone loss, measured as an off‐track injury, surgical treatment with the bone block procedure (Latarjet) was chosen. Due to the small dimension and pseudarthrosis of the coracoid process, a distal tibia allograft was used in addition to the coracoid graft.

### 2.1. Treatment

**Figure 2:**
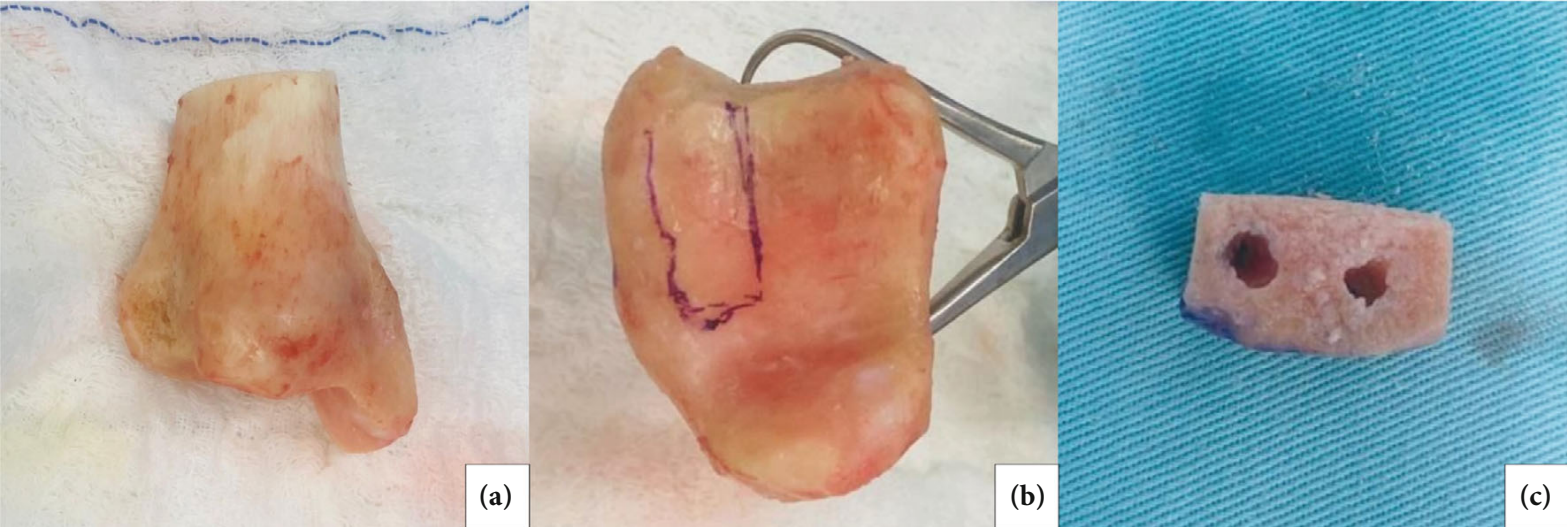
Graft preparation. (a) Whole piece of distal tibia allograft. (b) Articular view of the graft, showing the planned cut. (c) Final presentation of the graft after cutting and drilling for the screws.

**Figure 3:**
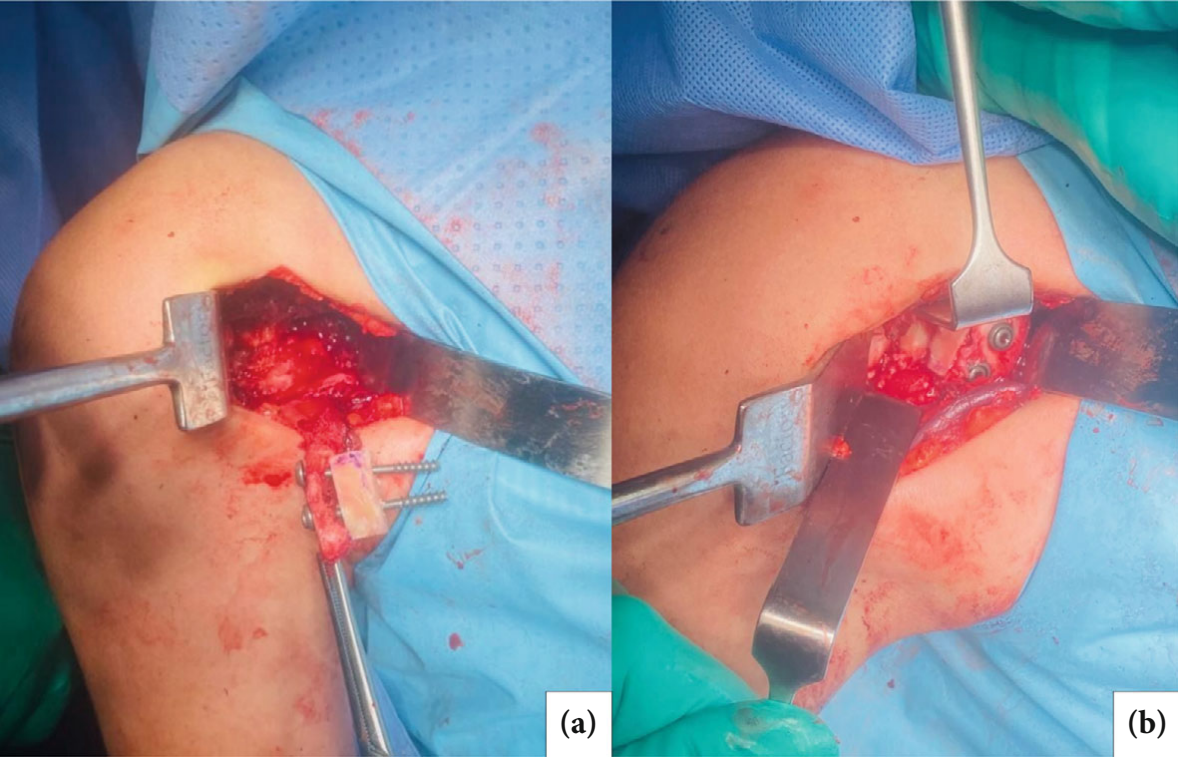
Patient in the beach chair position, deltopectoral incision. (a) Display of the combined graft assembly (coracoid on the left and distal tibia on the right) with screws. (b) Display of the graft after fixation onto the anterior glenoid.

The procedure was performed with the patient in the beach chair position under general anesthesia combined with regional nerve block. The surgical approach followed the technique described by Khundkar and Giele [7]. Intraoperatively, pseudarthrosis of the coracoid process was confirmed, and—as suspected during preoperative planning—its dimensions were insufficient to adequately restore the glenoid bone defect. A distal tibial allograft was therefore prepared to complement the reconstruction. The allograft was shaped to dimensions of 1.8 × 1.0 × 1.0 cm (length × width × depth) using a saw, and a drill guide was used to create two parallel holes (2.7 mm in diameter) to accommodate future screw fixation (Figure 2). Following preparation of both the glenoid surface and grafts (coracoid and allograft), the two grafts were aligned and temporarily assembled. This combined graft (autograft and allograft) was then fixed to the anteroinferior glenoid rim using two 3.5 mm cortical screws with washers, placed approximately 1 cm apart and centered on the graft (Figure 3), ensuring stable compression and anatomical positioning.

### 2.2. Postoperative Care

**Figure 4:**
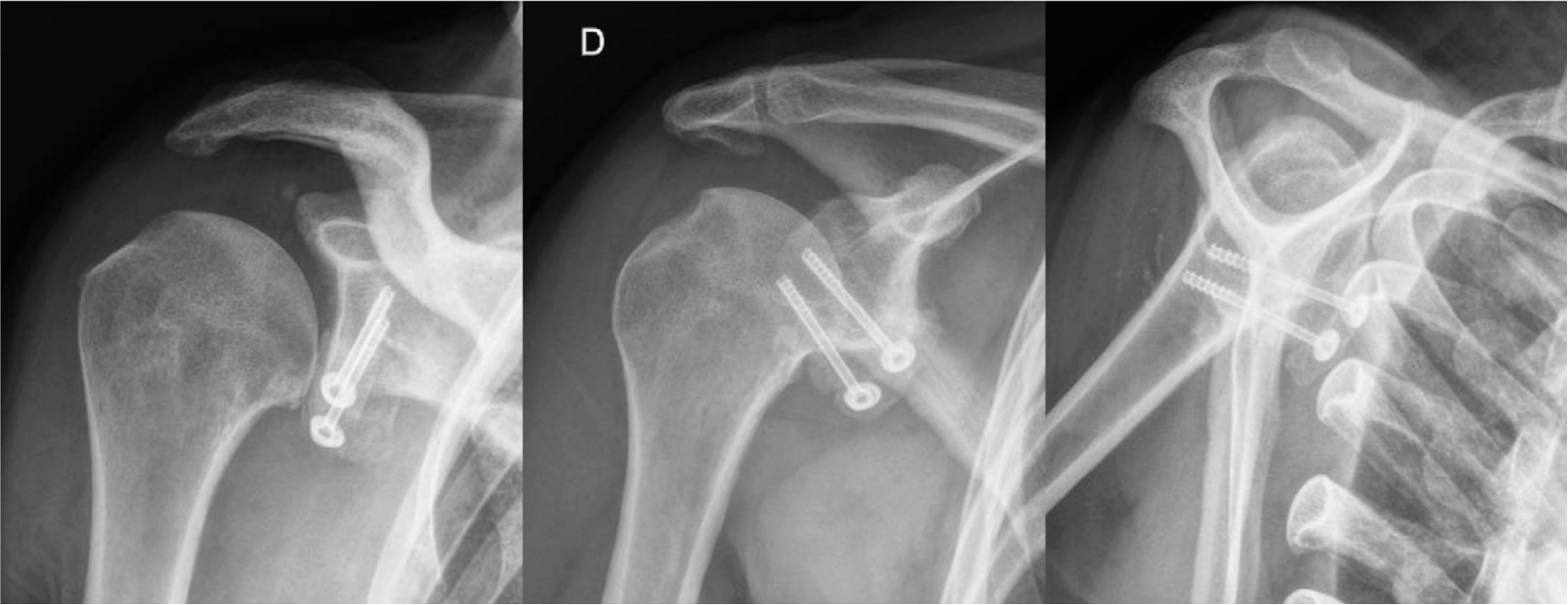
Anterior, oblique, and lateral views on radiographs of the right shoulder 2 months after surgery, showing good positioning of the graft and synthesis material, with early signs of consolidation.

The patient remained in a sling for 3 weeks to protect the surgery, performing only passive movements in physiotherapy to regain range of motion (ROM). After 3 weeks, the sling was removed, and active movements were allowed. Between 6 and 15 weeks, progressive strengthening was performed, and all activities were allowed at 16 weeks. At the end of rehabilitation, the patient achieved full recovery and functional ROM. Radiographs taken at 2 weeks and 2 months postoperatively showed well positioning of the graft and synthesis material and signs of consolidation and bone integration of the grafts with the glenoid (Figure 4).

**Figure 5:**
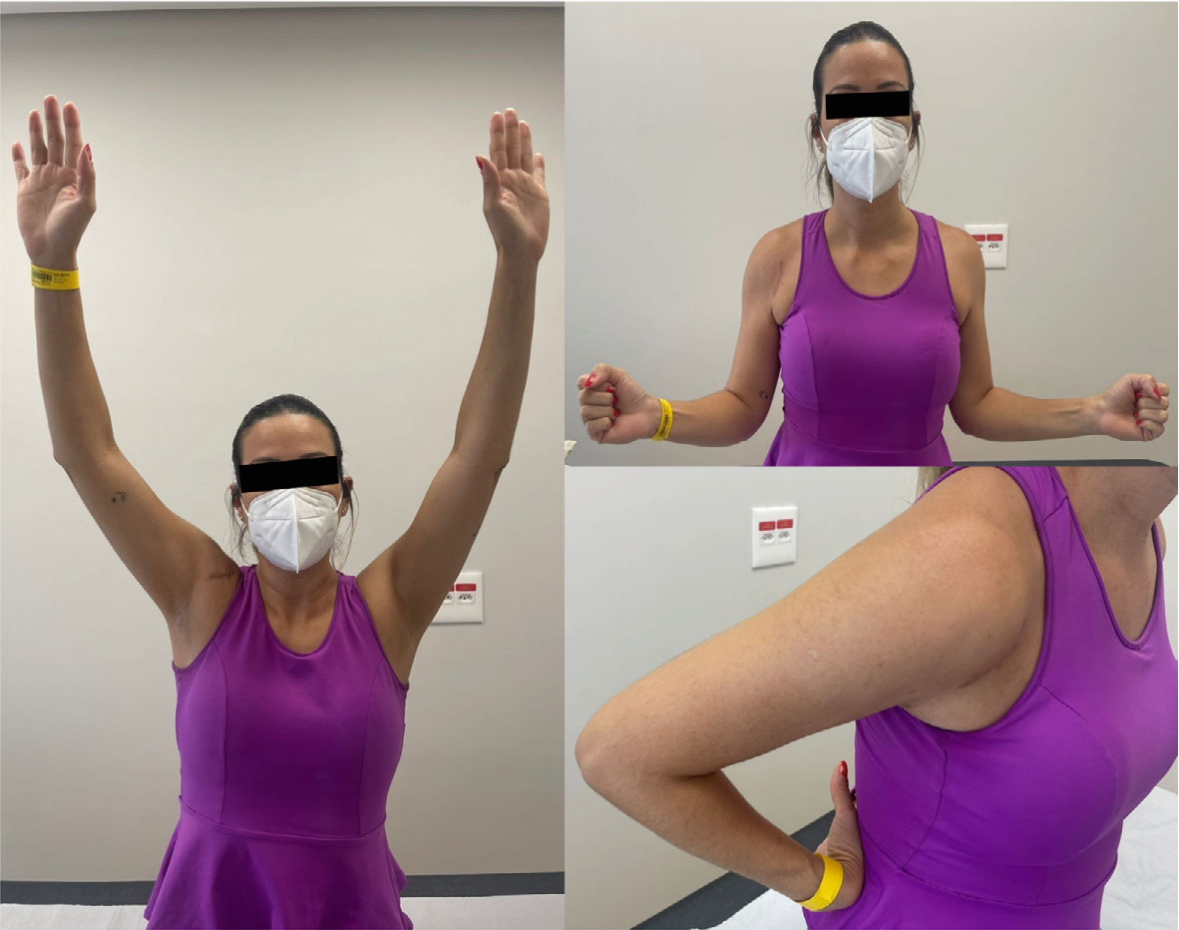
Clinical images of the patient 18 months after surgery, showing good recovery and functional range of motion.

At 18 months of follow‐up, the patient reported high satisfaction toward the treatment and recovery process. She has sustained no recurrences, presented with a negative apprehension test, maintained functional ROM (Figure 5), good functional scores (Figure 1 and Table 1), and reported mild pain only after intense overhead activities.

## 3. Discussion

Glenohumeral dislocation associated with seizures presents unique challenges due to high recurrence rates and significant forces during seizures [1]. The Latarjet technique, which uses the coracoid process to increase stability, has good outcomes in women [8], but has limitations, as highlighted by Ersen et al. [9]. In their study, epileptic patients who underwent the Latarjet procedure had a higher risk of redislocation (9%) compared to those who used distal tibia allograft (1.8%).

In our case, the patient′s coracoid was insufficient for adequate glenoid reconstruction, necessitating a complementary allograft due to significantly greater glenoid bone loss. The use of distal tibia allograft is an excellent alternative for significant glenoid bone loss cases, as highlighted by Liles et al. [10]. Additionally, the vascularization of the coracoid, primarily supplied by a direct branch from the second part of the axillary artery, is crucial for graft viability [7]. Preserving this vascularization is essential to avoid complications such as avascular necrosis and ensure graft integration.

The combined technique used in this case offers the advantage of addressing the extensive bone defect with an additional bone allograft—providing greater defect filling than coracoid transfer alone could achieve [11, 12]—while preserving the sling effect created by the conjoint tendon attached to the coracoid process [13]. Furthermore, the positioning of the screws and washers (two screws with washers were used, placed 1 cm apart and 0.7 cm from the graft apex) in this case was carefully planned with a focus on biomechanics, aiming to maximize torque strength and load to failure, as previously described [14].

Distal tibial allografts have demonstrated significant advantages in glenoid reconstruction, including precise anatomical restoration, a low risk of recurrent instability, favorable clinical outcomes, and minimal graft resorption [10, 15]. Notably, Dawe et al. reported that apparent graft resorption is, in fact, part of a physiological remodeling process that progressively restores native glenoid morphology [16]. Additionally, recent evidence reinforces the superiority of screw fixation for securing these allografts—particularly in revision settings—with studies showing low recurrence rates, improved functional outcomes, and reliable graft incorporation [17, 18]. Together, these findings support the validity of the combined approach adopted in the present case.

While the Latarjet procedure remains a reliable option for primary stabilization, its role in cases of high‐grade bone loss or for revision surgery is more controversial, as outcomes are generally inferior and recurrence rates are higher compared to simpler or primary cases [19]. In cases where the coracoid process is insufficient or has been previously transferred, distal tibial allograft has emerged as an effective alternative, providing a large osteochondral surface that restores the glenoid arc and articular congruity. This graft not only addresses extensive bone defects that exceed the reconstructive potential of a Latarjet but also offers biomechanical stability comparable to the native glenoid [20, 21]. Therefore, the combined use of Latarjet and distal tibial allograft may be considered in revision settings with high risk of failure, maximizing bone restoration while preserving the sling effect of the conjoint tendon for additional dynamic stability.

Furthermore, the clinical and radiographic outcomes of the Latarjet procedure versus glenoid reconstruction with distal tibia allograft, as discussed by Provencher et al. [3], suggest that both methods are effective, but the distal tibia allograft may offer a more durable solution with fewer complications [3, 9]. In this case, combining both grafts provided an effective solution for significant glenoid bone loss and Hill–Sachs lesion, allowing optimal functional recovery for the patient.

This case report is the first to document the combination of the Latarjet procedure with distal tibia allograft. However, the study has limitations: absence of objective functional evaluations, short follow‐up, and its methodology, which limits generalizability. Future research with a larger sample size and more robust methodology is necessary to assess long‐term outcomes and comparability with standard techniques.

## 4. Conclusion

The Latarjet procedure with distal tibia allograft augmentation is a viable alternative to the Latarjet procedure in patients with significant bone loss and coracoid pseudarthrosis. The technique provides joint stability and good mid‐term functional outcomes, offering a robust solution for the complex challenges of glenohumeral instability.

## Consent

Written informed consent was obtained from the patient prior to the induction of this study.

## Conflicts of Interest

The authors declare no conflicts of interest.

## Funding

No funding was received for this manuscript.

## Data Availability

The data that support the findings of this study are available from the corresponding author upon reasonable request.
